# Drug hypersensitivity in drug-resistant tuberculosis

**DOI:** 10.1016/j.waojou.2023.100778

**Published:** 2023-05-20

**Authors:** Zeynep Yegin Katran, İsmet Bulut, Aylin Babalık, Metin Keren, Fatma Merve Tepetam, Selver Seda Mersin, Cihan Örçen, Tuğçe Yakut, Dilek Yavuz

**Affiliations:** a Department of Allergy and Immunology, University of Health Sciences, Süreyyapaşa Training and Research Hospital, Istanbul, Turkey; b Department of Chest Diseases, University of Health Sciences, Süreyyapaşa Training and Research Hospital, İstanbul, Turkey; c Department of Allergy and Immunology, Dr Ersin Arslan Training and Research Hospital, Gazıantep, Turkey; d Department of Allergy and Immunology, Derınce Training and Research Hospital, Kocaeli, Turkey; e Department of Allergy and Immunology, Diyarbakır Training and Research Hospital, Diyarbakır, Turkey

**Keywords:** Drug resıstance tuberculosis, Urticaria, Maculopapular drug eruptıon, Pyrazınamıde

## Abstract

**Objective:**

To evaluate drug resıstant tuberculosis patients who developed drug hypersensitivity to antituberculosis drug.

**Methods:**

This was a retrospective study. The primary aim of the study is to determine the demographic and clinical characteristics of patients who develop drug hypersensitivity in drug resistant tuberculosis patients. The secondary aim of the study is to examine the treatment results. Demographic features, tuberculosis diagnostic indicator, clinical signs of developing hypersensitivity reaction, reaction time, and treatment were evaluated.

**Results:**

A total of 25 patients were included in the study. The prevalence of hypersensitivity in drug resistance patients was 11.9%. Twelve (48%) of the cases were women. Mean age (mean ± SD) was 37.24 ± 14.44 years; early type hypersensitivity reaction in 13 (52%). Three patients were isoniazid resistant; 19 patients were multidrug-resistant (MDR); 2 patients were pre-extensive drug resistant (Pre-XDR), 1 patient was extensive drug resistance (XDR) tuberculosis. The most common skin findings were maculopapular eruption and urticaria. But also we had seen ısole angıodema, urtıcarıa and angıoedema, erythema multıforme, lıchenoıd drug eruptıon and drug rash with eosinophilia and systemic symptoms. In patients who developed a hypersensitivity reaction, the responsible agent was identified in 14 cases in total. Among the drugs, pyrazinamide, ethambutol, moxifloxacin, amikacin, para amino salicylic, prothionamide, and cycloserine are the responsible agents. When evaluated in terms of treatment results, 15 (60%) patients successfully completed the treatment.

**Conclusion:**

Our study is the first study in the literature that evaluated the drug hypersensitivity in drug resıstance tuberculosis patients. Drug hypersensitivity that develops with tuberculosis treatment may lead to discontinuation or change in treatment. İt can cause treatment failure, drug resistance, relapse, and even death. In resistant tuberculosis, the already existing resistance pattern may become more difficult to treat. Success can be achieved with the right management in these patients who have few treatment options, more drug side effects, and high treatment failure rates. The established regimen should be curative and prevent recurrence.

## Introduction

Tuberculosis is the most common infectious agent after COVID-19 infection worldwide.^1^ Although tuberculosis is a treatable and preventable disease, drug resistance is encountered due to delayed diagnosis and errors in the application of anti-tuberculosis treatment.^2^ Adverse drug reactions impair treatment compliance.^3^ In susceptible patients, the regimen is composed of isoniazid, rifampicin, ethambutol, and pyrazinamide; In resistant cases, it consists of a combination of many drugs such as additional quinolones, aminoglycosides, linezolid, bedaqulin, dalamanid, ethionamide.^4^ One of the conditions affecting the success of tuberculosis treatment is drug hypersensitivity. We have little information on treatment outcomes in multidrug-resistant (MDR) tuberculosis. In the meta-analysis, which includes 50 studies from 25 countries, treatment success is 61%. The use of linezolid, fluoroquinolones, bedaqulin, clofazamine, and carbapenem is associated with treatment success.^5^ When hypersensitivity develops in the treatment of tuberculosis, proper management is important in the success of treatment. If hypersensitivity is managed correctly, a drug that is necessary for the success of treatment becomes available to patients. According to Sharma et al, the pattern of resistance is associated with regular and uninterrupted intake of antituberculosis drugs.^6^ It was also observed that resistant tuberculosis patients who developed adverse drug reactions had lower quality of life scores and worse adherence to treatment.^7^

It is crucial to manage drug hypersensitivity reactions correctly in patients with drug-resistant tuberculosis to ensure that they receive appropriate and effective treatment. If the hypersensitivity reaction is not managed appropriately, it can lead to treatment failure and disease progression, which can have serious consequences for the patient's health. In our country, treatment of resistant tuberculosis is carried out in 4 centers. One of these centers is our hospital. Our patients used drugs including bedaqulin and dalamanid in different combinations. In our study, which is the first case series to examine drug hypersensitivity in resistant tuberculosis patients, demographic, clinical characteristics, and treatment management of patients were examined.

## Material – methods

The design of the study was a case series, carried out between 01.02.2015 and 01.05.2021. Patients aged 18 years and older who developed immediate-type and delayed-type hypersensitivity due to antituberculosis drug and were consulted to the Allergy and Immunology clinic were examined. The primary aim of the study is to determine the demographic and clinical characteristics of patients who develop hypersensitivity in resistant tuberculosis patients. The secondary aim of the study is to examine the treatment results.

Demographic data of the patients, diagnosis of tuberculosis, clinical features of hypersensitivity reaction, and time of occurrence, drug treatments, and treatment results were evaluated. Age, gender, and nationality were noted in demographic data. According to the World Health Organization (WHO), countries were evaluated in 6 different regions according to their level of development. These were Africa, the Americas, the Eastern Mediterranean, Europe, Asia, and the Western Pacific.^1^ In our study, cases were also analyzed according to WHO country grouping.

Tuberculosis diagnoses, organ involvement, and treatments were evaluated as determined in the Turkish Ministry of Health Tuberculosis Diagnosis and Treatment Guide published in 2019.^8^ Diagnoses were classified as smear positive, culture positive, molecular test positive, histopathological diagnosis, and clinical radiological diagnosis; organs affected from tuberculosis were classified as pulmonary and extrapulmonary; the extrapulmonary group was grouped as miliary, lymph node, pleura, kidney, pericardium, and larynx.^8^ Resistances were grouped as isoniazid resistance MDR tuberculosis, pre-extensive drug resistance (Pre-XDR), and extensive drug resistance (XDR). In tuberculosis treatment, resistance to both isoniazid and rifampicin is called MDR tuberculosis. Resistance to a quinolone or parenteral drug in addition to MDR is called pre-extensive drug resistance (Pre-XDR) tuberculosis. Resistance to a quinolone and parenteral drug in addition to MDR is called extensive drug resistance (XDR) tuberculosis.^1^^,^^8^

Hypersensitivity reactions have been evaluated as defined in the 2019 Approach to Drug Hypersensitivity Reactions: National Guideline Update and EAACI guideline.^9^^,^^10^ When the type 1 immediate drug hypersensitivity reaction was mentioned as urticaria, angioedema, rhinitis, conjunctivitis, bronchospasm, abdominal pain, syncope, anaphylaxis, which develops in the first hour after taking the drug but could last up to 6 h, were evaluated. Type 4 hypersensitivity reactions were evaluated as maculopapular eruption, fixed drug eruption, Steven Johnson Syndrome (SJS), Toxic Epidermal Necrolysis (TEN), acute generalized eczematous pustulosis (AGEP), drug reaction with eosinophilia and systemıc symptoms (DRESS), erythema multiforme, and lichenoid drug eruption that developed 6 h or later after taking the drug.

Patients who developed a hypersensitivity reaction were treated first. After all symptoms had resolved, the drugs were given by desensitization one-by-one. The way of desensitization is given by the protocol made by Katran et al.^11^ In immediate type hypersensıtıvıty, each drug was given in at least 6 steps; 2.5 times between doses and drug administration times not exceeding 30 min. The drugs were given under the supervision of a physician and nurse. A drug was given every day. If hypersensitivity did not develop, the next day after the full dose of the drug was given, the new drug was added by desensitization. In delayed type hypersensıtıvıty, drugs were given in the same doses as the early type, with desensitization. The new drug was added to the treatment 4 days later. We did not use premedication.

In the statistics of the study, all analyses were performed using SPSS 22.0.

## Results

During the study, 210 drug resistance patients were hospitalized in the Tuberculosis inpatient service. A total of 25 patients were included in the study. The prevalence of hypersensitivity in drug resistance patients was 11.9%. Twelve (48%) of the cases were women. Mean age (mean ± SD) was 37.24 ± 14.44 years; immediate type hypersensitivity reaction in 13 (52%) cases; 16 (64%) of them were citizens of the Republic of Turkey; 24 (96%) of them were diagnosed bacteriologically; 21 (84%) had pulmonary tuberculosis; 5 (20%) of them had previously received antituberculosis treatment. Three patients were isoniazid resistant; 19 patients were rifampicin resistant/MDR; 2 patients were pre-XDR, 1 patient was XDR tuberculosis (Table 1).

**Table 1:** Demographic and clinical characteristics of the patients

VARIABLE			N (%)
**GENDER**	**FEMALE**		12 (%48)
	**MALE**		13 (%52)
**AGE**	**(MEAN ± SD)**		37.24 ± 14.44
**HYPERSENSITIVITY REACTION TYPE**	**IMMEDIATE TYPE**		13 (%52)
	**DELAYED TYPE**		12 (%48)
**NATIONALITY**	**TURKEY**		16 (%64)
	**OTHER**	**RUSSIA**	3 (%12)
		**UZBEKISTAN**	1 (%4)
		**TURKMENISTAN**	1 (%4)
		**AZERBAIJAN**	1 (%4)
		**KYRGYZSTAN**	2 (%8)
		**SYRIA**	1 (%4)
**WHO COUNTRY CLASSIFICATION**		**ASIA**	22 (%88)
		**EUROPE**	3 (%12)
		**AFRICA**	–
		**AMERICA**	–
		**EASTERN MEDITERRANEAN**	–
		**WESTERN PACIFIC**	–
**DIAGNOSIS**		**SPUTUM POSITIVE**	18 (%72)
		**CULTURE POSITIVE**	6 (%24)
		**MOLECULAR TEST POSITIVE**	1 (%4)
**ORGAN AFFECTED FROM TUBERCULOSİS**	**PULMONARY**		21 (%84)
	**EXTRAPULMONARY**	**MILIARY**	1 (%4)
		**LYMPH NODE**	1 (%4)
		**KIDNEY**	1 (%4)
		**PERICARDIUM**	1 (%4)
		**PLEURA**	–
		**BONE**	–
		**LIVER**	–
**PRIOR TREATMENT**	**NO**		20 (%80)
	**YES**	**RECCURENCE**	2 (%8)
		**PATIENT OUT OF FOLLOW UP**	1 (%4)
		**TREATMENT FAILURE**	2 (%8)
**RESISTANCE**		**ISONIAZID RESISTANCE**	3 (%12)
		**MDR**	19 (%76)
		**PRE-XDR**	2 (%8)
		**XDR**	1 (%4)

The most common skin findings were maculopapular eruption and urticaria. But also we had seen ısole angıodema, urtıcarıa and angıoedema, erythema multıforme, lıchenoıd drug eruptıon and DRESS (Table 2).

**Table 2:** Clinical features and treatment outcomes of patients.

Case	Clinical Features of Hypersensitivity	Hypersensitivity Management	Culprit Drug	Inıtıal Regımen	Fınal Regımen	Duration Without Treatment	Treatment Outcomes
1	Urtıcarıa	Treated with antihistamines and steroids	–	Moxı+ Cyc + Pza + Clph + Lzd + Amikacin	Moxı+Cyc + Pza + Clph + Lzd + Amikacin	12 days	Completed
2	Urtıcarıa	Self resolved, tolerated desensitization	–	Moxı+ Cyc + Pza + Clph + Lzd + Amikacin + Eth	Moxı+ Cyc + Pza + Clph + Lzd + Amikacin + Eth	8 days	Completed
3	Urtıcarıa	Treated with antihistamines	–	Inh + Moxi + Proth + Emb + Pza	Inh + Moxi + Proth + Emb + Pza	11 days	Completed
4	Urtıcarıa	Treated with antihistamines	–	Emb + Pza + Proth + PAS + Cyc	Moxi + PAS + Lzd + Dlm + Bdq	13 days	Completed
5	Urtıcarıa + Angıoedema	Treated with antihistamines	–	Moxi + Rif + Amikacin + Proth + Cyc	Moxi + Rif + Amikacin + Proth + Cyc	18 days	Completed
6	Urtıcarıa	Self resolved, tolerated desensitization	–	Moxi + Cyc + Proth + Lzd + PAS	Moxi + Cyc + Proth + Lzd + PAS	8 days	Completed
7	Urtıcarıa	Treated with antihistamines and steroids	Moxı, PAS	Amikacin + Cyc + Moxi + PAS + Pza + Proth	Amikacin + Cyc + Pza + Proth	9 days	Completed
8	Angıoedema	Treated with antihistamines	–	Amikacin + Cyc + Emb + Moxi + Proth + PAS + Pza	Amikacin + Cyc + Moxi + PAS + Lzd	14 days	Completed
9	Urtıcarıa	Treated with antihistamines and steroids	Cyc	Inh + Emb + Moxi + Proth + Pza + Cyc	Inh + Emb + Moxi + Proth + Pza + PAS	32 days	Still
10	Urtıcarıa	Self resolved, tolerated desensitization	–	Moxi + Amikacin + Cyc + Emb + Pza	Moxi + Lzd + Cyc + Emb + Pza	4 days	Completed
11	Angıoedema	Treated with antihistamines	Emb Pza	Inh + Rif + Emb + Pza	Inh + Rif + Moxi	15 days	Completed
12	Urtıcarıa	Treated with steroids	–	Moxi + Amikacin + Cyc + Emb + Pza + Proth	Moxi + Amikacin + Cyc + Emb + Pza + Proth	12 days	Still
13	Urtıcarıa + Angıoedema	Self resolved, tolerated desensitization	–	Amikacin + Cyc + Moxi + PAS	Amikacin + Cyc + Moxi + PAS	3 days	Still
14	MPE	Self resolved, tolerated desensitization	–	Emb + Proth + Cyc + Pza + PAS + Moxi + PAS	Emb + Proth + Cyc + Pza + PAS + Moxi + PAS	15 days	Transferred
15	MPE	Treated with antihistamines and steroids	–	Moxi + Amikacin + Cyc + Proth + Pza + PAS	Moxi + Amikacin + Cyc + Proth + Pza + Lzd	28 days	Completed
16	MPE	Treated with antihistamines	–	Pza + PAS + Moxi + Proth + Cyc + Lzd + Dlm + Bdq	Pza + PAS + Moxi + Proth + Cyc + Lzd + Dlm + Bdq	36 days	Still
17	MPE	Self resolved, tolerated desensitization	–	Amikacin + Cyc + Moxi + PAS + Proth + Pza	Amikacin + Cyc + Moxi + PAS + Proth + Pza	28 days	Transferred
18	DRESS	Treated with antihistamines and steroids	Emb Pza Moxı, Proth, Cyc	Amikacin + Cyc + Moxi + PAS + Proth + Emb + Pza	Levo + Lzd + Amikacin + PAS	55 days	Transferred
19	MPE	Treated with antihistamines and steroids	Amikacin, PAS	Amikacin + Cyc + Moxi + PAS + Proth + Pza	Cyc + Moxi + Proth + Pza	56 days	Still
20	MPE	Treated with antihistamines	–	Amikacin + Emb + Lzd + Clph + PAS + Pza + Moxi	Amikacin + Emb + Lzd + Clph + PAS + Pza + Moxi	22 days	Lost
21	MPE	Treated with antihistamines	Pza	Inh + Emb + Moxi + Pza + Strep	Amikacin + Emb + Proth + Cyc + Levo	24 days	Completed
22∗	Erythema Multiforme	Treated with antihistamines and steroids	–	Inh + Rif + Emb + Pza	Inh + Rif + Emb + Pza	34 days	Still
23	MPE	Treated with antihistamines and steroids	–	Moxi + PAS + Cyc	Moxi + PAS + Cyc	23 days	Completed
24	MPE	Treated with antihistamines and steroids	Amikacin	Amikacin + Moxi + PAS + Pza + Cyc + Proth	Moxi + PAS + Pza + Cyc + Proth + Lzd + Clph	49 days	Completed
25	Lichenoid Drug Eruption	Treated with antihistamines and steroids	–	Moxi + Cyc + Amikacin + Proth + PAS	Moxi + Cyc + Amikacin + Proth + PAS	51 days	Completed

**F:** female, **M:** male, **DRESS:** Drug rash with eosinophilia and systemic symptoms, **MPE:** Maculopapular Drug Eruption, **Emb:** Ethambutol, **Eth:** Ethionamide, **Moxı:** Moxifloxacin, **PAS:** para amino salicylic acid, **Cyc:** Cyloserıne, **Emb:** Ethambutol, **Pza:** Pyrazinamide, **Proth:** prothionamide, **Lzd:** Linezolid, **Dlm:** Dalamanid, **Bdq:** Bedaqulın, **Clph:** clophazamine, **Inh:** Isonıazıd, **Rif:** Rifampicin, **Levo:** Levofloxacin, **Strep**: Streptomycin. **Treatment Outcomes: Completed:** Succesfully Completed the Treatment. **Still:** Still Under Treatment. **Lost:** Lost to follow up **Transferred:** Transferred abroad. ∗HIV posıtıve.

When we evaluated the tıme to develop ımmedıate- and delayed-type hypersensıtıvıty together; ıt (mean ± SD) was 21.6 ± 28.45 days. After the development of drug hypersensitivity, only tuberculosis treatment was discontinued in 6 (24%) patients and patients were self resolved and tolerated desensitization. Antihistamine treatment was given in 8 (32%) patients, oral/parenteral steroid was given in 1 (4%) patient, and antihistaminic and oral/parenteral steroid were given together in 10 (40%) patients.

In patients who developed a hypersensitivity reaction, the responsible agent was identified in 14 cases in total with skın prıck test, ıntradermal test, and patch test. Among the drugs, pyrazinamide, ethambutol, moxifloxacin, amikacin, PAS, prothionamide, and cycloserine are the responsible agents (Table 3).

**Table 3:** Drugs responsible for hypersensitivity

Responsıble Drug	Patients who developed hypersensitivity	Total Number of Patients Using Drug
Isonıazıde	–	5
Rıfampıcıne	–	3
Etanbutol	2	13
Pyazınamıde	3	20
Streptomycın	–	1
Levofloxacıne	–	2
Moxıfloxacıne	2	24
Capreoycıne	–	–
Amıkasıne	2	16
Para amınosalıcylıc asıd	2	15
Protıonamıde	1	16
Cycloserıne	2	20
Lınezolıde	–	11
Total	14	25

The duration of treatment was 18.57 ± 4.91 months (10–25 months). When evaluated in terms of treatment results, 15 (60%) patients successfully completed the treatment; 1 (4%) patient could not be reached; 3 (12%) patients were transferred abroad, and 6 (24%) patients are still under treatment.

## Discussion

Tuberculosis is an infectious disease that requires long treatment and patient compliance. Moreover, if there is drug resistance, it is more difficult for both doctors and patients. Adverse events, including hypersensitivity, result in poor adherence and treatment success.^12^^,^^13^ In this study, patients who developed immediate- or delayed-type drug hypersensitivity with drug-resistant tuberculosis treatment were evaluated. Immediate-delayed type reaction was seen with similar frequency. The most common skin findings were urticaria and maculopapular eruption. Treatment success was also reported as 60%.

There are 4 centers in our country that hospitalize patients with drug-resistant tuberculosis. Two of them are in Istanbul. Our center is a reference hospital and has the highest number of beds in Istanbul. In our study the prevalence of hypersensitivity in drug-resistant patients was 11.9%. The prevalence of drug hypersensitivity has been reported to be between 4.4 and 13%, similiar to our study.^14^, ^15^, ^16^ However, this prevalence was higher than drug-sensitive tuberculosis patients.^17^

Drug hypersensitivity was observed with similar frequency in men and women. There is no gender difference in drug hypersensitivity in patients with resistant tuberculosis.^14^ On the other hand, there were studies indicating that it was more common in women.^18^ Prospective studies with large numbers of patients necessary.

In a study conducted in Uganda, patients who developed resistant tuberculosis were examined. The patient age range was seen as 25–44 years. In our study, the patient age range was similar.^19^ The mean age of the patients was found to be similar in many studies.^14^^,^^17^

Many more and different drug regimens are used in MDR, pre-XDR, and XDR tuberculosis. The incidence of adverse events was not higher in isoniazid-resistant patients than in drug-sensitive patients.^18^ Second-generation antituberculosis drugs used in resistant patients were associated with more adverse events and poor treatment outcomes.^17^^,^^20^ Considering the drugs responsible for hypersensitivity, a wide variety was remarkable. But when we look at the literature, we can mention that each agent is likely to be responsible for hypersensitivity. The risk of adverse events was lower with quinolones, bedaqulin, and clofazimine but higher with the second line injectable drugs, aminosalicylic acid, and linezolid.^21^ In the study of Tan et al, the most frequently responsible agent was pyrazinamide (performed in drug-sensitive patients).^17^ Our study was conducted in resistant patients and the number of alternative agents was much higher. Although the number of patients was small, pyrazinamide was the most responsible agent for hypersensitivity.

Immediate- and delayed-type reaction was seen with similar frequency. The most common skin findings were maculopapular eruption and urticaria. Although number of patients were small, there were severe reactions such as ertyhema multıforme and DRESS. The drug causing erythema multiforme could not be determined. The agents responsible for the development of DRESS were ethambutol, pyrazinamide, moxifloxacin, prothionamide and cycloserine. In the study of Tan et al, the most common delayed type reaction and maculopapular drug eruption were reported. However, it was also mentioned that all dermatological findings can be seen like urtıcarıa, exfoliative dermatitis, lichenoid eruption.^17^ In the literature, there are case examples of DRESS, AGEP, SJS, urtıcarıa, and Lichenoid drug eruption in drug-resistant tuberculosis patients.^22^, ^23^, ^24^, ^25^

In our study, if the patients needed systemic steroids or antihistamines until their rash resolved, they were given for treatment. Both interrupting the treatment and adding steroids is a situation that doctors fear in terms of resistance and treatment failure. It was observed that the risk of death increased 3 times when the treatment was interrupted in the initial phase.^26^

In this situation, where the treatment option has decreased, such as resistant tuberculosis, this is a complete nightmare. Therefore, we recommend resuming drug therapy very quickly.

We think that it is correct to give each drug individually by desensitization. The desensitization scheme we used is the same as in the study of Katran et al. Due to the limited choice of drugs to be used in the treatment of tuberculosis, drugs that cause hypersensitivity were given primarily by desensitization. If hypersensitivity developed again while desensitization was applied, the drug was changed.

Our recommendation;

1. Give the drugs one by one.
2. Give with desensitization
3. Wait one or some days for each drug.
4. Not change the whole regimen when hypersensitivity develops
5. Focus on the last drug you added to the treatment.

Already, changing the responsible agent is compatible with better treatment outcomes than changing all drugs.^25^

Our study is the first case series in the literature that evaluated the drug hypersensitivity in drug resıstant tuberculosis patients. Drug hypersensitivity that develops with tuberculosis treatment may lead to discontinuation or change in treatment. İt can cause treatment failure, drug resistance, relapse and even death. In resistant tuberculosis, the already existing resistance pattern may become more difficult to treat.^27^ Success can be achieved with the right management in these patients who have few treatment options, more drug side effects, and high treatment failure rates. The established regimen should be curative and prevent recurrence.

The management of drug-resistant tuberculosis with drug hypersensitivity reactions requires a multidisciplinary approach and should be carried out in experienced centers. Managing drug hypersensitivity reactions in patients with drug-resistant tuberculosis is a complex process that requires the expertise of several healthcare providers, including infectious disease specialists, pulmonologists, allergists/immunologists, and pharmacists. These specialists can work together to ensure that the patient receives appropriate and effective treatment while minimizing the risk of adverse reactions.

## Abbreviation

AGEP, Acute generalized eczematous pustulosis; Cyc, cycloserine; DRESS, drug rash with eosinophilia and systemic symptoms; Emb, Ethambutol; Moxi, moxifloxacin; MDR, Multidrug-resistant; Pre-XDR, Pre-extensive drug resistance; PAS, Para amınosalıcylıc asıd; Pza, Pyrazinamide; Proth, prothionamide; SJS, Steven Johnson Syndrome; TEN, Toxic Epidermal Necrolysis; XDR, Extensive drug resistance.

## Acknowledgements

I would like to express my special thanks of gratitude to my mum and my dad.

## Funding

This research did not receive any specific grant from funding agencies in the public, commercial, or not-for-profit sectors.

## Availability of data and materials

Data available on request from the authors.

## Authors' contributions

All authors take part of Conceptualization; Data curation; Formal analysis; Investigation; Methodology; Validation; Visualization; Writing-original draft.

## Ethics approval and consent to participate

Ethics committee approval of the University of Health Sciences, Süreyyapaşa Chest Diseases and Thoracic Surgery Training and Research Hospital was obtained for this study. Written informed consent to participate and publish was obtained from all individual participants included in the study.

## Author's consent for publication

All authors reviewed and approved the final version for publication in World Allergy Organization Journal.

## Declaration of competing interest

The authors have no conflicts of interest to declare.
